# Comparison of left ventricular mechanics in runners versus bodybuilders using speckle tracking echocardiography

**DOI:** 10.1186/s12947-015-0002-y

**Published:** 2015-02-18

**Authors:** Ipoly Szauder, Attila Kovács, Gábor Pavlik

**Affiliations:** Cardiologic Diagnostic Centre, Szabó Ilonka Str. 31, H-1015 Budapest, Hungary; Cardiovascular Imaging Research Group, Heart and Vascular Center, Semmelweis University, Budapest, Hungary; University of Physical Education, Budapest, Hungary

**Keywords:** Athlete’s heart, Runners, Bodybuilders, Speckle tracking, Strain

## Abstract

**Background:**

Athlete’s heart is a common definition for a broad spectrum of adaptations induced by intense exercise. We intended to compare left ventricular (LV) mechanics in two sports disciplines with different exercise nature: marathon runners (endurance) and bodybuilders (power).

**Methods:**

24 marathon or ultramarathon runners (R), 14 bodybuilders (B) and 15 healthy, sedentary male volunteers (N) were investigated. Beyond standard echocardiographic protocol, parasternal short-axis and apical recordings optimized for speckle tracking analysis were acquired (Esaote MyLab 25). Using dedicated software (TomTec 2D Performance Analysis), global longitudinal (GLS), circumferential (GCS) and radial strain (GRS) were calculated by averaging the corresponding 16 LV segments. Data are presented as mean ± SD.

**Results:**

Calculated LV mass was higher in bodybuilders compared to normal controls (R vs. B vs. N: 198 ± 52 vs. 224 ± 69 vs. 186 ± 30 g, p < 0.05). We found no difference regarding conventional systolic function parameters among the groups (ejection fraction: 55 ± 9 vs. 60 ± 6 vs. 59 ± 5%; mitral lateral S’ velocity: 10.7 ± 0.6 vs. 10.6 ± 0.4 vs. 11.0 ± 0.8 cm/s). However, speckle tracking analysis showed a different pattern of myocardial deformation in our groups: while GRS was similar, GLS was decreased in runners, GCS was decreased in bodybuilders compared to the other two groups (GLS: -19.4 ± 3.4 vs. -23.3 ± 2.1 vs. -24.1 ± 3.0; GCS: -26.6 ± 3.8 vs. -22.4 ± 4.3 vs. -26.4 ± 2.7%, p < 0.05). Significant correlations were found in runners between GLS and end-diastolic volume (r = 0.46; p < 0.05), and body surface area (r = 0.49; p < 0.05). In bodybuilders, GCS was closely related to LV mass (r = 0.61; p < 0.01) and systolic blood pressure (r = 0.42; p < 0.05).

**Conclusions:**

While conventional morphological and functional echocardiographic parameters failed to distinguish between the athlete’s heart of the two different sport disciplines, deformation parameters showed a different pattern of LV mechanics in runners versus bodybuilders.

**Electronic supplementary material:**

The online version of this article (doi:10.1186/s12947-015-0002-y) contains supplementary material, which is available to authorized users.

## Background

Athlete’s heart is a common expression for the various adaptive changes induced by intense exercise [1-5]. However, it has been widely investigated that the type and intensity of the training, age or gender all significantly influence the cardiovascular adaptation and can lead to morphological and functional differences among athletes [2,6]. Therefore, athlete’s heart can be interpreted only in its distinct context.

Traditionally, sport disciplines can be divided by their exercise nature to endurance or power groups [2,4,7]. Although this oversimplification is barely useful, the endpoints of this scale can be identified: marathon-ultramarathon runners train and race on extreme long distances which results in pure endurance strain, while bodybuilders perform mainly static work aiming to increase muscle mass [8,9]. Nowadays, both sport gained unexpected popularity, raising clinical questions regarding the induced adaptive (or maladaptive) changes in the cardiovascular system.

The complex helical myofiber architecture of the left ventricle (LV) is of great clinical interest since deformation data in multiple directions provide valuable information in numerous pathological and physiological conditions [10]. The novel method of speckle tracking echocardiography allows us to quantify this mechanical aspect of the LV performance and also to investigate the different types of deformation separately.

Our aim was to investigate the cardiac adaptation of marathon runners and bodybuilders using speckle tracking echocardiography. We hypothesized that different deformation pattern exists in these sport disciplines.

## Subjects and methods

In this study highly trained male athletes were included: 24 marathon or ultramarathon runners and 14 bodybuilders. Fifteen healthy, sedentary male volunteers were also enrolled. Subjects in the athlete groups had an at least 5 year history of regular exercise and trained at least 11 months a year. The regime of marathon runners consisted of 20.0 ± 1.4 training hours/week for 7.8 ± 1.6 years, while of bodybuilders 16.8 ± 1.1 hours/week for 7.9 ± 1.4 years in average. Marathoners covered at least 50 kilometres a week and also participating in professional competitions. Bodybuilders trained at least five times a week. Healthy volunteers had no more than three hours regular physical activity a week. Exclusion criteria were any acute or chronic disease, use of any medication or performance-enhancing drugs, or suspicion of presence of any cardiovascular disease based on blood pressure measurements, ECG, laboratory tests or echocardiography. Our protocol was designed in accordance to the ethical principles of the Declaration of Helsinki and all participants provided their written informed consent before entering the study. The protocol was approved by the Semmelweis University Institutional Committee for Science and Research Ethics (TUKEB 175/2012).

**Figure 1:**
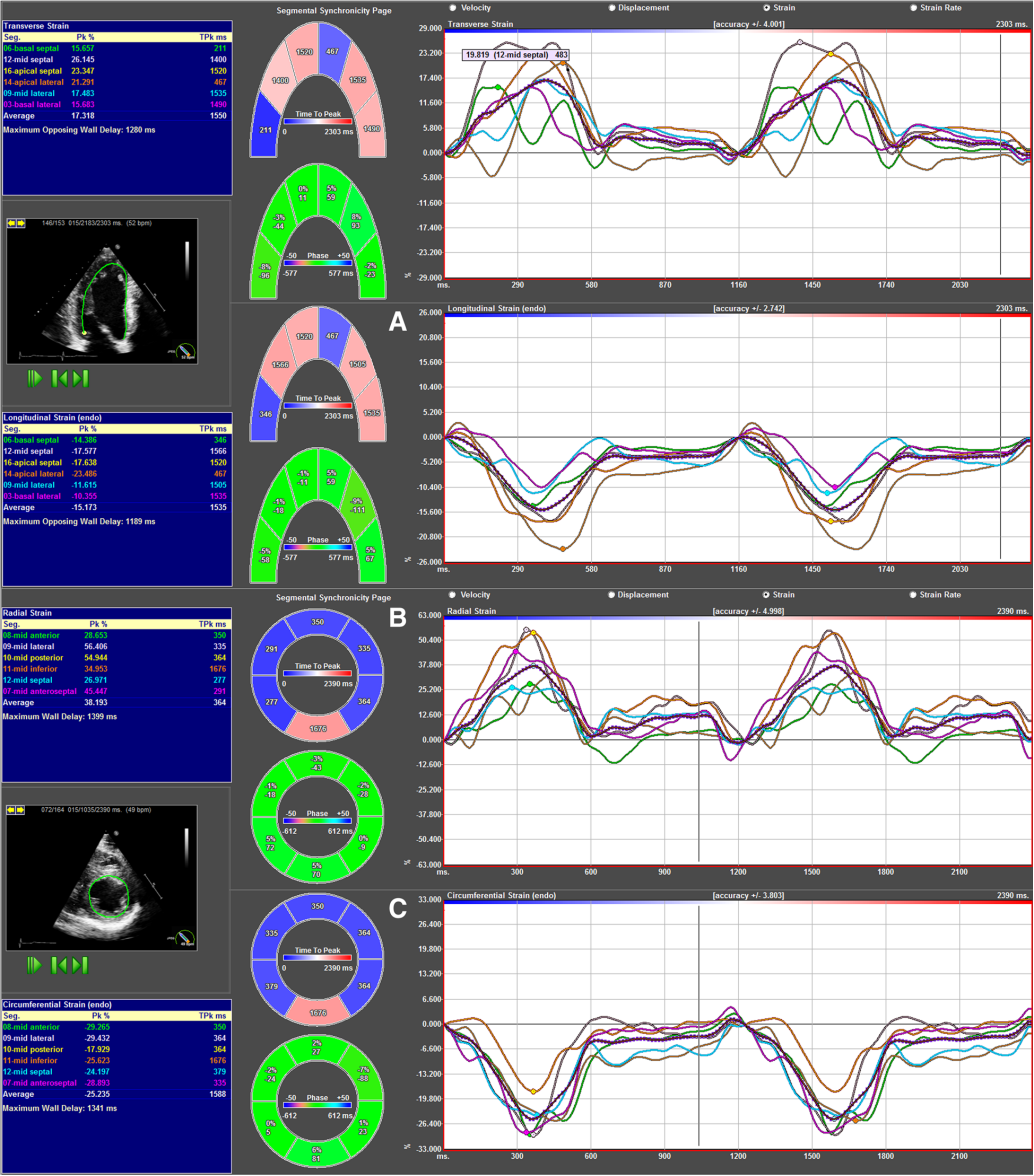
**Representative layout of the speckle tracking analysis in an athlete.** Using the apical four-chamber view (upper part) and the midpapillary level of the parasternal short-axis view (lower part), negative values of longitudinal strain **(A)**, positive values of radial strain **(B)** and negative values of circumferential strain **(C)** of the corresponding segments can be obtained.

Before the ultrasonic assessment, detailed medical history was obtained, then subjects underwent physical examination, basic anthropometric and blood pressure measurements (office and 24 h ambulatory blood pressure monitoring as well) on the basis of current guidelines [11]. Standard laboratory tests were also performed. Echocardiography was performed using an Esaote Biomedica MyLab 25 system equipped with a 2.5-3.5 MHz phased-array transducer (Esaote S.p.A, Genoa, Italy). Conventional two-dimensional, M-mode, spectral Doppler and tissue Doppler measurements were performed according to current guidelines [12,13]. LV mass was calculated using the Devereux formula [14]. Exclusion criteria were: presence of wall motion abnormalities, at least moderate valvular disorders or diastolic dysfunction suggested by Doppler measurements. Beyond standard echocardiographic protocol, loops optimized for speckle tracking analysis were also acquired (parasternal short-axis views at mitral valve, midpapillary and apical levels, apical four-, three- and two-chamber views). LV myocardium was optimally visualized by adequate settings of gain, focus, depth and sector width to reach a frame rate over 60 frames per second. All echocardiographic examinations were performed by the same experienced operator (IS). Speckle tracking analysis was performed using a dedicated software (2D Cardiac Performance Analysis, TomTec Imaging Systems GmbH, Unterschleissheim, Germany; Figure 1) by a user with remarkable expertise of the software environment (AK). LV endocardial borders were delineated manually and corrected, if the software failed to track them precisely throughout the cardiac cycle. Segmental peak systolic strain values were averaged over 2 cardiac cycles. To calculate global longitudinal, circumferential and radial peak systolic strain, corresponding segmental values of the standard 16 LV segments were used [15]. Body surface area (BSA) was calculated using the Mosteller formula [16]. Standard BSA indexed values of LV volumes and mass were presented, however, this calculation implies some limitations based on data from the literature and also own experience [17,18].

For statistical analysis we used Statistical Package for the Social Sciences software version 20 (IBM, Armonk, New York, USA). Normal distribution of variables was assessed by Shapiro-Wilk test. To compare variables, one-way ANOVA followed by Fisher post-hoc test was used. Relationships were calculated with Pearson correlation test. Data are presented as mean ± SD. P values under 0.05 were considered statistically significant.

## Results

Basic characteristics of the study groups are summarized in Table 1. There was no difference in age and height, however, bodybuilders had higher weight, in line with body surface area and body mass index. Heart rate and systolic and diastolic blood pressures were lower in runners compared to the other groups.

**Table 1:** **Basic characteristics of the study groups**

	***Runner (R)***	***Bodybuilder (B)***	***Normal control (N)***	***p < 0.05***	***p < 0.01***	***p < 0.001***
*n*	24	14	15			
*age (years)*	27 ± 3	27 ± 3	27 ± 3			
*height (cm)*	182 ± 6	179 ± 6	180 ± 5			
*weight (kg)*	77 ± 9	90 ± 20	82 ± 9	B > N		B > R
*BSA (m* ^*2*^ *)*	1.9 ± 0.1	2.1 ± 0.2	2.0 ± 0.1	B > N	B > R	
*BMI (kg/m* ^*2*^ *)*	23 ± 2	28 ± 5	25 ± 3		B > N	B > R
*HR (bpm)*	63 ± 8	72 ± 8	74 ± 4			R < B,N
*SBP (mmHg)*	122 ± 4	132 ± 9	133 ± 4			R < B,N
*DBP (mmHg)*	75 ± 7	87 ± 4	87 ± 4			R < B,N

One-way ANOVA followed by Fisher post-hoc test. Data are presented as mean ± SD. B: bodybuilders, BMI: body mass index, bpm: beats per minute, BSA: body surface area, DBP: diastolic blood pressure, HR: heart rate, N: normal controls, R: runners, SBP: systolic blood pressure.

Table 2 contains the echocardiographic data of the groups. We found no wall motion abnormalities, any moderate or severe valvular, acute or chronic myocardial or pericardial disease in the subjects. Diastolic septal and lateral wall thicknesses were similar, however, the calculated LV mass was higher in the bodybuilder group compared to controls. Both type of athletes had higher LV end-diastolic volumes. No difference in the systolic function assessed by ejection fraction or by tissue Doppler imaging of the mitral annulus could be detected.

**Table 2:** **Echocardiographic data of the study groups**

	***Runner (R)***	***Bodybuilder (B)***	***Normal control (N)***	***p < 0.05***	***p < 0.01***	***p < 0.001***
*IVSd (mm)*	10.8 ± 1.4	11.3 ± 1.7	10.6 ± 0.9			
*LVIDd (mm)*	49.8 ± 4.8	51.4 ± 4.8	48.2 ± 3.8	B > N		
*LVPWd (mm)*	10.5 ± 1.3	10.9 ± 1.3	10.6 ± 0.9			
*LVM (g)*	198 ± 52	224 ± 69	186 ± 30	B > N		
*LVMi (g/m* ^*2*^ *)*	100 ± 24	105 ± 24	92 ± 15			
*EDV (mL)*	176 ± 32	178 ± 36	157 ± 26	R,B > N		
*EDVi (mL/m* ^*2*^ *)*	85 ± 16	84 ± 12	77 ± 12			
*ESV (mL)*	80 ± 24	72 ± 17	69 ± 16			
*ESVi (mL/m* ^*2*^ *)*	37 ± 12	34 ± 7	33 ± 8			
*EF (%)*	55 ± 9	60 ± 6	59 ± 5			
*septal S’ (cm/s)*	8.4 ± 0.5	8.7 ± 1.6	8.6 ± 0.7			
*lateral S’ (cm/s)*	10.7 ± 0.6	10.6 ± 0.4	11.0 ± 0.8			
*longitudinal strain (%)*	−19.4 ± 3.4	−23.3 ± 2.1	−24.1 ± 3.0	R < B,N		
*circumferential strain (%)*	−26.6 ± 3.8	−22.4 ± 4.3	−26.4 ± 2.7	B < R,N		
*radial strain (%)*	42.5 ± 5.5	44.2 ± 8.2	44.1 ± 4.5			

One-way ANOVA followed by Fisher post-hoc test. Data are presented as mean ± SD. The more negative results represent the greater extent of longitudinal and circumferential deformation. B: bodybuilders (n = 14), EDV: end-diastolic volume, EF: ejection fraction, ESV: end-systolic volume, IVS: interventricular septal thickness, LVID: left ventricular internal diameter, LVM: left ventricular mass, LVPW: left ventricular posterior wall thickness, N: normal controls (n = 15), R: runners (n = 24). “d” refers to end-diastolic measures, “i” for indexed values to body surface area.

**Figure 2:**
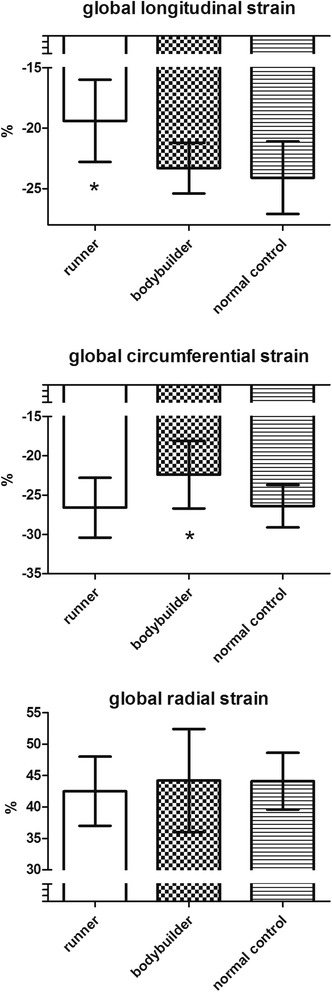
**Comparison of deformation parameters between runners (n = 24), bodybuilders (n = 14) and normal controls (n = 15).** *p < 0.05 vs. the other two groups.

However, speckle tracking analysis showed a different pattern of myocardial deformation between the groups. While radial strain was similar, longitudinal strain was lower in runners, circumferential strain was lower in bodybuilders compared to the other groups (Figure 2 and Additional file 1; the more negative results represent the greater extent of longitudinal and circumferential deformation).

We found significant correlations in runners between longitudinal strain and end-diastolic volume (r = 0.46; p < 0.05), end-diastolic volume index (r = 0.43; p < 0.05) and body surface area (r = 0.49; p < 0.05). In bodybuilders, circumferential strain was closely related to LV mass (r = 0.61; p < 0.01), mass index (r = 0.46; p < 0.05) and systolic blood pressure (r = 0.42; p < 0.05).

## Discussion

We aimed to compare two very different sport disciplines in terms of myocardial mechanics: highly trained marathon runners and bodybuilders. While conventional echocardiographic parameters failed to be different between the two groups, speckle tracking derived strain indices showed a different pattern of deformation compared to each other and to healthy controls as well.

The multiple helical fiber orientation of the LV is a well known phenomenon [19], however, the contribution of separate deformation directions to LV performance is a novel field of interest. Longitudinal strain refers to the shortening of the LV in long-axis (negative value), and has become a valuable clinical parameter which reflects numerous pathological conditions (i.e. ischemia) sensitively [20]. Circumferential strain can be interpreted by a short-axis shortening of the myofibers (negative value) and has been shown to be the major deformation direction generating ejection [21]. Radial thickening on a short-axis image is a summation of other deformation directions, which can be quantified by radial strain (positive value). Different pathologies can alter uniquely this deformation pattern [22,23], therefore we hypothesized that sport disciplines with divergent exercise nature can also result in different adaptation regarding myocardial mechanics.

Athlete’s heart is characterized by a physiological increase in LV mass [1-4]. A classical hypothesis suggests that sports with mainly endurance exercise nature (long-distance running, cycling, etc.) result in excentric LV hypertrophy, while power sports (weightlifting) induce concentric hypertrophy [7]. However, the spectrum of athlete’s heart is very broad and substantive investigation of the adaptation induced by mostly endurance or power training is difficult. Therefore, we selected our study population to address this issue. Long-distance running is a good example of clear endurance training, while the goal of bodybuilders is completely different with weightlifting: to provide spectacular outlook and huge amount of muscle mass. To date, no study has investigated this two very popular sports head-to-head.

In contrast to our expectations, the groups of athletes were similar to each other regarding morphological features provided by echocardiography. Furthermore, conventional parameters of LV systolic function (ejection fraction, S’ by tissue Doppler imaging) also failed to distinguish between the two types of athlete’s heart.

The novel method of speckle tracking echocardiography allows us to easily characterize myocardial mechanics on standard two-dimensional grey-scale images [24]. We found that longitudinal strain is lower in endurance athletes, while this decrease correlated with the increase in LV end-diastolic volume and also body surface area. These associations can be explained by the phenomenon of a larger LV that needs less deformation to maintain stroke volume during resting conditions. Furthermore, lower baseline strain values with preserved ejection fraction may also imply a possible functional reserve capacity and could be a sign of a trained heart. On the contrary, Simsek and coworkers found increased longitudinal strain in athletes while comparing eccentric and concentric types of LV hypertrophy [25]. The possible explanation can be the significantly lower age of their population, as the age-related decrease of longitudinal strain has been reported [26]. It also highlights the issue of proper selection of the athletes’ groups which can lead to the interpretation of inconsistent results in the literature [27,28].

In bodybuilders the circumferential strain was found to be lower. In this group, the decrease in circumferential shortening strongly correlated with increased LV mass. The relationship between increased LV mass and decreased strain values is well-known from previous studies involving patients with hypertrophic cardiomyopathy, aortic stenosis, and also with hypertension-induced hypertrophy [29-32]. Based on this and also on the correlation between systolic blood pressure and circumferential strain values in bodybuilders, suspicion of pathological remodelling may be reasonable despite our study design was remarkably aimed to exclude arterial hypertension in advance. Thus, our results better represent preliminary, cross-sectional data, as we are following-up our athletes regularly and looking for further clinical relevance of the strain parameters.

In-depth analysis of myocardial mechanics raises the question whether strain parameters could be able to differentiate pathological and physiological remodelling and maybe could help to monitor the training phases of an athlete. Promising data are available regarding a classical dilemma of sports medicine: differentiation between hypertrophic cardiomyopathy and athlete’s heart [2,33,34]. Nevertheless, if we would like to use speckle tracking to help sport-specific training we have to understand the deformation profile of a given discipline. Aiming that, larger studies are needed, however, our data may help to highlight this issue.

## Limitations

Obvious limitations of our study are the relatively low case number and its cross-sectional approach. We used 2D speckle tracking analysis, however, the added value of 3D strain has been reported recently [35]. Recruiting only ultramarathoners in the runners group could be beneficial, but would result in a significant drop regarding sample size. Despite the strong denial of the subjects, use of any drug cannot be ruled out.

## Conclusions

While conventional morphological and functional echocardiographic parameters failed to distinguish between the athlete’s heart of two different sport disciplines, strain parameters showed a different pattern of LV deformation in runners versus bodybuilders. Longitudinal strain is lower in runners, whilst circumferential strain is decreased in bodybuilders. Speckle tracking analysis can be useful for identifying previously undetected differences regarding athlete’s heart.

## Additional file

Additional file 1:
**Representative comparison of deformation pattern in a runner (left side) versus a bodybuilder (right side).** Using the apical four-chamber view (upper part) and the midpapillary level of the parasternal short-axis view (lower part), longitudinal strain, radial strain and circumferential strain of the corresponding segments can be obtained. In a representative pair of athletes, global longitudinal strain (GLS) was lower in the runner (left side), while global circumferential strain (GLS) was lower in the bodybuilder (right side).

## Abbreviations

2D: Two-dimensional
3D: Three-dimensional
B: Bodybuilders
BSA: Body surface area
ECG: Electrocardiogram
GCS: Global circumferential strain
GLS: Global longitudinal strain
GRS: Global radial strain
LV: Left ventricular
N: Normal controls
R: Runners
SD: Standard deviation

## References

[1] Pavlik G, Major Z, Varga-Pinter B, Jeserich M, Kneffel Z (2010). The athlete's heart Part I (Review). Acta Physiol Hung.

[2] Pelliccia A, Maron BJ (1997). Outer limits of the athlete's heart, the effect of gender, and relevance to the differential diagnosis with primary cardiac diseases. Cardiol Clin.

[3] Utomi V, Oxborough D, Whyte GP, Somauroo J, Sharma S, Shave R (2013). Systematic review and meta-analysis of training mode, imaging modality and body size influences on the morphology and function of the male athlete's heart. Heart.

[4] Pluim BM, Zwinderman AH, van der Laarse A, van der Wall EE (2000). The athlete's heart. A meta-analysis of cardiac structure and function. Circulation.

[5] Kovacs A, Olah A, Lux A, Matyas C, Nemeth BT, Kellermayer D, et al. Strain and strain rate by speckle tracking echocardiography correlate with pressure-volume loop derived contractility indices in a rat model of athlete's heart. Am J Physiol Heart Circ Physiol 2015:ajpheart 00828 02014. http://www.ncbi.nlm.nih.gov/pubmed/25617359 10.1152/ajpheart.00828.2014 25617359

[6] Pavlik G, Major Z, Csajagi E, Jeserich M, Kneffel Z (2013). The athlete's heart. Part II: influencing factors on the athlete's heart: types of sports and age (review). Acta Physiol Hung.

[7] Morganroth J, Maron BJ, Henry WL, Epstein SE (1975). Comparative left ventricular dimensions in trained athletes. Ann Intern Med.

[8] Predel HG (2014). Marathon run: cardiovascular adaptation and cardiovascular risk. Eur Heart J.

[9] Ahlgrim C, Guglin M (2009). Anabolics and cardiomyopathy in a bodybuilder: case report and literature review. J Card Fail.

[10] Mondillo S, Galderisi M, Mele D, Cameli M, Lomoriello VS, Zaca V (2011). Speckle-tracking echocardiography: a new technique for assessing myocardial function. J Ultrasound Med.

[11] Mancia G, Fagard R, Narkiewicz K, Redon J, Zanchetti A, Bohm M (2013). ESH/ESC guidelines for the management of arterial hypertension: the Task Force for the Management of Arterial Hypertension of the European Society of Hypertension (ESH) and of the European Society of Cardiology (ESC). Eur Heart J.

[12] Lang RM, Bierig M, Devereux RB, Flachskampf FA, Foster E, Pellikka PA (2005). Recommendations for chamber quantification: a report from the American Society of Echocardiography's Guidelines and Standards Committee and the Chamber Quantification Writing Group, developed in conjunction with the European Association of Echocardiography, a branch of the European Society of Cardiology. J Am Soc Echocardiogr.

[13] Nagueh SF, Appleton CP, Gillebert TC, Marino PN, Oh JK, Smiseth OA (2009). Recommendations for the evaluation of left ventricular diastolic function by echocardiography. Eur J Echocardiogr.

[14] Devereux RB, Reichek N (1977). Echocardiographic determination of left ventricular mass in man. Anatomic validation of the method. Circulation.

[15] Cerqueira MD, Weissman NJ, Dilsizian V, Jacobs AK, Kaul S, Laskey WK (2002). Standardized myocardial segmentation and nomenclature for tomographic imaging of the heart. A statement for healthcare professionals from the Cardiac Imaging Committee of the Council on Clinical Cardiology of the American Heart Association. Circulation.

[16] Mosteller RD (1987). Simplified calculation of body-surface area. N Engl J Med.

[17] George KP, Gates PE, Whyte G, Fenoglio RA, Lea R (1999). Echocardiographic examination of cardiac structure and function in elite cross trained male and female Alpine skiers. Br J Sports Med.

[18] Pavlik G, Olexo Z, Frenkl R (1996). Echocardiographic estimates related to various body size measures in athletes. Acta Physiol Hung.

[19] Sengupta PP, Korinek J, Belohlavek M, Narula J, Vannan MA, Jahangir A (2006). Left ventricular structure and function: basic science for cardiac imaging. J Am Coll Cardiol.

[20] Feigenbaum H, Mastouri R, Sawada S (2012). A practical approach to using strain echocardiography to evaluate the left ventricle. Circ J.

[21] Altekin RE, Kucuk M, Yanikoglu A, Karakas MS, Er A, Ozel D (2012). Evaluation of the left ventricular regional function using two-dimensional speckle tracking echocardiography in patients with end-stage renal disease with preserved left ventricular ejection fraction. Acta Cardiol.

[22] Kovacs A, Tapolyai M, Celeng C, Gara E, Faludi M, Berta K (2014). Impact of hemodialysis, left ventricular mass and FGF-23 on myocardial mechanics in end-stage renal disease: a three-dimensional speckle tracking study. Int J Cardiovasc Imaging.

[23] Ernande L, Bergerot C, Girerd N, Thibault H, Davidsen ES, Gautier Pignon-Blanc P (2014). Longitudinal myocardial strain alteration is associated with left ventricular remodeling in asymptomatic patients with type 2 diabetes mellitus. J Am Soc Echocardiogr.

[24] Leitman M, Lysyansky P, Sidenko S, Shir V, Peleg E, Binenbaum M (2004). Two-dimensional strain-a novel software for real-time quantitative echocardiographic assessment of myocardial function. J Am Soc Echocardiogr.

[25] Simsek Z, Hakan Tas M, Degirmenci H, Gokhan Yazici A, Ipek E, Duman H (2013). Speckle tracking echocardiographic analysis of left ventricular systolic and diastolic functions of young elite athletes with eccentric and concentric type of cardiac remodeling. Echocardiography.

[26] Kaku K, Takeuchi M, Tsang W, Takigiku K, Yasukochi S, Patel AR (2014). Age-related normal range of left ventricular strain and torsion using three-dimensional speckle-tracking echocardiography. J Am Soc Echocardiogr.

[27] Schattke S, Xing Y, Lock J, Brechtel L, Schroeckh S, Spethmann S (2014). Increased longitudinal contractility and diastolic function at rest in well-trained amateur Marathon runners: a speckle tracking echocardiography study. Cardiovasc Ultrasound.

[28] Monte IP, Mangiafico S, Buccheri S, Bottari VE, Lavanco V, Arcidiacono AA, et al: Myocardial deformational adaptations to different forms of training: a real-time three-dimensional speckle tracking echocardiographic study. Heart and vessels 2014. http://www.ncbi.nlm.nih.gov/pubmed/?term=24820450 10.1007/s00380-014-0520-9 24820450

[29] Sengupta SP, Caracciolo G, Thompson C, Abe H, Sengupta PP (2013). Early impairment of left ventricular function in patients with systemic hypertension: new insights with 2-dimensional speckle tracking echocardiography. Indian Heart J.

[30] Galderisi M, Esposito R, Schiano-Lomoriello V, Santoro A, Ippolito R, Schiattarella P (2012). Correlates of global area strain in native hypertensive patients: a three-dimensional speckle-tracking echocardiography study. Eur Heart J Cardiovasc Imaging.

[31] Urbano-Moral JA, Rowin EJ, Maron MS, Crean A, Pandian NG (2014). Investigation of global and regional myocardial mechanics with 3-dimensional speckle tracking echocardiography and relations to hypertrophy and fibrosis in hypertrophic cardiomyopathy. Circulation Cardiovascular imaging.

[32] Staron A, Bansal M, Kalakoti P, Nakabo A, Gasior Z, Pysz P (2013). Speckle tracking echocardiography derived 2-dimensional myocardial strain predicts left ventricular function and mass regression in aortic stenosis patients undergoing aortic valve replacement. Int J Cardiovasc Imaging.

[33] Kovacs A, Apor A, Nagy A, Vago H, Toth A, Nagy AI (2014). Left ventricular untwisting in athlete's heart: key role in early diastolic filling?. Int J Sports Med.

[34] Butz T, van Buuren F, Mellwig KP, Langer C, Plehn G, Meissner A (2011). Two-dimensional strain analysis of the global and regional myocardial function for the differentiation of pathologic and physiologic left ventricular hypertrophy: a study in athletes and in patients with hypertrophic cardiomyopathy. Int J Cardiovasc Imaging.

[35] Stefani L, De Luca A, Toncelli L, Pedrizzetti G, Galanti G (2014). 3D Strain helps relating LV function to LV and structure in athletes. Cardiovasc Ultrasound.

